# Activation of Wnt signaling in human fracture callus and nonunion tissues

**DOI:** 10.1016/j.bonr.2024.101780

**Published:** 2024-06-19

**Authors:** Michael Hadjiargyrou, Maria Kotsiopriftis, Dominique Lauzier, Reggie C. Hamdy, Peter Kloen

**Affiliations:** a Department of Biological & Chemical Sciences, New York Institute of Technology, Old Westbury, NY 11568, USA; b Division of Orthopaedic Surgery, Shriners Hospital for Children, Montreal Children Hospital, McGill University, Montreal, QC H4A 0A9, Canada; c Department of Orthopedic Surgery and Sports Medicine, Amsterdam UMC, location Meibergdreef 9, Amsterdam, the Netherlands; d Amsterdam Movement Sciences, (Tissue Function and Regeneration), Amsterdam, the Netherlands

**Keywords:** Wnt, Nonunion, Fracture, Callus, Hypertrophic, Oligotrophic

## Abstract

The Wnt signaling pathway is a key molecular process during fracture repair. Although much of what we now know about the role of this pathway in bone is derived from in vitro and animal studies, the same cannot be said about humans. As such, we hypothesized that Wnt signaling will also be a key process in humans during physiological fracture healing as well as in the development of a nonunion (hypertrophic and oligotrophic). We further hypothesized that the expression of Wnt-signaling pathway genes/proteins would exhibit a differential expression pattern between physiological fracture callus and the pathological nonunion tissues. We tested these two hypotheses by examining the mRNA levels of key Wnt-signaling related genes: ligands (WNT4, WNT10a), receptors (FZD4, LRP5, LRP6), inhibitors (DKK1, SOST) and modulators (CTNNB1 and PORCN). RNA sequencing from calluses as well as from the two nonunion tissue types, revealed that all of these genes were expressed at about the same level in these three tissue types. Further, spatial expression experiments identified the cells responsible of producing these proteins. Robust expression was detected in osteoblasts for the majority of these genes except SOST which displayed low expression, but in contrast, was mostly detected in osteocytes. Many of these genes were also expressed by callus chondrocytes as well. Taken together, these results confirm that Wnt signaling is indeed active during both human physiological fracture healing as well as in pathological nonunions.

## 1 Introduction

Fracture healing is a complex physiologic process that involves the coordinated activities of several cell types, guided by various signaling pathways (Hadjiargyrou et al., 2002; Hadjiargyrou and O'Keefe, 2014). The healing process is roughly divided into four phases: hematoma and inflammation, callus formation (osteogenesis, chondrogenesis), endochondral ossification and finally remodeling (Hadjiargyrou and O'Keefe, 2014). In rodent models, fracture repair generally takes about 21 days for the bones to have consolidated. In adult humans, it takes about 6–12 weeks depending on the fracture location and age and/or comorbidities of the patient. Once healed, the new bone is indistinguishable from the surrounding tissue, representing a true regenerative process. This process closely recapitulates embryonic bone development, without the inflammatory phase (Hadjiargyrou and O'Keefe, 2014).

Despite ongoing technical advances in operative fracture treatment (such as new plates and screws, surgical navigation and 3D-CT pre-operative planning) and the rapidly increasing knowledge of the molecular and cellular mechanisms involved in fracture healing, an estimated 2 to 5 % of fractures do not heal properly and go on to become a nonunion (Marsh, 1998). The current more pragmatic definition of a nonunion is a fracture that will not heal without surgical intervention. A nonunion has an enormous socioeconomic impact on the patient's life with high psychological stresses for both, the patient and his/her family. Recent studies have quantitated the large impact of a long bone nonunion on the quality of life (Schottel et al., 2015; Brinker et al., 2017).

Of the various pathways involved in bone regeneration and fracture healing, the Bone Morphogenetic Protein (BMP) and the Wnt/β-catenin signaling pathways are the most well studied. We previously reported on the role and expression of BMP in human fractures and nonunions (Kloen et al., 2002; Kloen et al., 2012). While there are many animal studies on the activation of the Wnt/β-catenin, pathway during fracture repair, including from our lab (Hadjiargyrou et al., 2002; Zhong et al., 2006), there are very few studies using human callus and nonunions. Long et al. (2019) performed a microarray analysis to identify differentially expressed miRNAs in human atrophic nonunion tissues and further downstream experiments found that miR-381 modulated osteogenic differentiation via regulating β-catenin by binding to the 3′UTR of Wnt5A and FZD3. Hofmann et al. (2008) found that osteoblasts isolated from human nonunions exhibited reduced cell viability, differentiation and accompanied by significant downregulation of genes involved in the canonical Wnt signaling pathway. Alterations in Wnt/β-catenin signaling can also lead to impaired bone healing and skeletal dysplasia (e.g. van Buchem disease, sclereostosis, osteosclerosis) (Chang et al., 2014).

Similar to BMP signaling, the Wnt pathway is highly conserved (Steinhart and Angers, 2018). Wnt proteins are a family of 19 secreted cysteine-rich glycoproteins. The Wnt proteins become palmitoylated in the endoplasmatic reticulum in the presence of Porcupine (PORCN). The modified Wnt proteins are then secreted and bind to plasma membrane receptors (Frizzled, FZD), and their co-receptors, low-density lipoprotein receptor protein (LRP), 5 or 6. The binding of Wnt results in activation of the phosphoprotein Disheveled (DSH), which inhibits a cytoplasmic complex of glycogen synthase-kinase 3 (GSK-3) to finally inhibit the inactivation of β-catenin (CTNNB1). β-catenin is then released and stabilized in the cytoplasm and subsequently translocates to the nucleus to initiate transcription of Wnt-target genes. Dickkopf (DKK) and secreted Frizzled-related protein (sFRP) inhibit β-catenin signaling by blocking the interaction of the LRP5/6 and FZD receptors. Sclerostin (SOST) is another secreted Wnt-antagonist that interacts with LRP5/6 to prevent binding of Wnt ligands (Holdsworth et al., 2012). Further, there are three main pathways for intracellular Wnt signaling: canonical, also known as the Wnt/β-catenin pathway, and two non-canonical pathways, the planar cell polarity (PCP) pathway and the Wnt/Ca^++^ pathway (Wang et al., 2014). But by far, the best-known pathway is the canonical Wnt, in which ubiquination and degradation of β-catenin is inhibited.

The aim of the current study was to examine the mRNA and protein expression of key members of the Wnt-signaling pathway in human fracture callus and two different types of nonunion tissues (hypertrophic and oligotrophic). As such, we hypothesized that Wnt signaling will also be a key process in humans during physiological fracture healing as well as in the development of a nonunion (hypertrophic and oligotrophic). We further hypothesized that the expression of Wnt-signaling pathway genes/proteins would exhibit different expression pattern between physiological fracture callus and the pathological nonunion tissues. We tested these hypotheses by examining the mRNA levels of key Wnt-signaling related genes and by determining their (protein) spatial expression in physiological fracture calluses and nonunion tissues.

## 2 Material and methods

### 2.1 Specimens

Long bone (femur, humerus) fracture calluses (representing normal physiological healing callus, *n* = 10) and non-union tissue, hypertrophic, (HNU, *n* = 8) and oligotrophic (ONU, n = 10) was obtained during operative treatment of patients. A nonunion was defined as a fracture that would not heal without surgical intervention. The nonunions were classified according to the Weber and Çech classification (Weber and Cech, 1976). Table 1 shows the patient data and tissue characteristics. Briefly, the fracture calluses were harvested from five femoral and five humeri fractures. The average age of these patients (7 males and 3 females) was 37.9 yr (range 6–57). Non-union tissue was obtained during revision surgery of a HNU of the femur (*n* = 4) or humerus (n = 4). The average age of these patients (6 males and 1 females) was 62.6 yr. (range 37–80). Non-union tissue was also obtained during revision surgery of an ONU of the femur (*n* = 5), or the humerus (n = 5) and the average age of these patients (3 males, 7 females) was 52.7 (range 33–77). None of the non-unions was classified as atrophic. The “age” of the fracture callus (time since fracture) was an average 5.8 weeks (range 2–10). Time to nonunion tissue harvest was on an average of 15.3 months (range 5–47) for the HNU and 17.9 months (range 4–72) for the ONU. There was no infection based on intra-operatively taken deep tissue cultures. Given the small number of bone samples for each type, it was difficult to stratify or subset the samples by age, sex, time of fracture, etc. Unfortunately, this is a limitation with working with human samples. A fellowship trained orthopedic trauma surgeon (PK) treated all patients. All fractures and nonunions uneventfully healed after (revision) surgery. Consent for removal of the tissue and its storage in the tissue bank in a coded fashion for research purposes was obtained from each patient per Institutional Review Board (IRB) guidelines (W20_075 #20.103, Academic Medical Center, Amsterdam, the Netherlands).

**Table 1:** Patient/tissue characteristics.

Tissue type	Age/Sex	Location	Time since fracture	Co-morbidities
Fracture callus (Callus)	56/F	Humerus	10 w	None
	35/M	Femur	2.5 w	None
	28/M	Humerus	3 w	None
	44/M	Femur	4 w	Not obtained
	39/M	Femur	8 w	Not obtained
	20/M	Femur	4 w	Not obtained
	6/M	Femur	8 w	Not obtained
	40/F	Humerus	10 w	None
	57/F	Humerus	2 w	Hypothyroidism
	54/M	Humerus	6 w	None
Hypertrophic nonunion (HNU)	47/F	Humerus	16 mo	Mild asthma
	54/M	Femur	47 mo	None
	72/M	Humerus	11 mo	None
	74/M	Femur	9 mo	Diabetes mellitus, polyneuropathy, prostate cancer
	80/M	Femur	13 mo	None
	56/F	Humerus	20 mo	None
	74/M	Humerus	5 mo	Minor ischemic stroke
	37/M	Femur	11 mo	None
Oligotrophic nonunion (ONU)	62/F	Humerus	4 mo	None
	33/F	Humerus	9 mo	None
	53/F	Femur	9 mo	None
	39/F	Femur	36 mo	None
	55/F	Humerus	10 mo	Hypertension, horseshoe kidney, breast cancer
	50/M	Femur	10 mo	None
	39/M	Humerus	5 mo	None
	50/M	Femur	72 mo	Hypertension
	69/F	Femur	7.5 mo	COPD/hypertension
	77/F	Humerus	6 mo	Diabetes mellitus

Abbreviations: w = weeks; mo = months; M = male; F = female.

### 2.2 RNA sequencing

The human samples, patient characteristics and RNA extraction and sequencing (GEO dataset GSE226568) have been previously reported by La Manna et al. (2023) as well as by our laboratory (Salichos et al., 2024). From these data, we have used a total of 23 RNA seq samples for our analyses; physiological fracture callus (*n* = 6), HNU (*n* = 8), and ONU (*n* = 9). Although all of the Wnt-related genes were present in these analyses, we chose 2–3 genes as representatives for each category of: ligands (WNT4, WNT10a), receptors (FZD4, LRP5, LRP6), inhibitors (DKK1, SOST) and modulators (CTNNB1 and PORCN). As such, we only present detailed data on the expression of these select genes for all of the analyses.

### 2.3 Goldner's trichrome staining

Tissue specimens were processed similarly to previous studies conducted in our laboratory (Hadjiargyrou et al., 2000; Zhi et al., 2001; Lombardo et al., 2004; Komatsu and Hadjiargyrou, 2004; Gersch et al., 2005; Komatsu et al., 2007; Abdelmagid et al., 2010; Hadjiargyrou et al., 2023). Briefly, tissue specimens were fixed in 10 % neutral buffered formalin for 24h and subsequently decalcified in 10 % ethylenediamine tetra acetic acid (EDTA), pH 7.2 for 3 weeks, and then embedded in paraffin. Six micrometer sections were cut using a Leica RM 2255 microtome (Leica Microsystems, Richmond Hill, ON, Canada). Following deparaffinization and hydration, sections were stained by Goldner Trichrome using standard staining procedures. Photomicrographs of the tissues were taken using a Leica microscope (Leica Microsystems, Richmond Hill, ON, Canada) attached to a Q-Imaging camera (Olympus DP70, Concord, ON, Canada).

### 2.4 Immunohistochemistry

Directly after collecting, the callus and nonunion tissues were fixed in 10 % neutral buffered formalin for 24 h and subsequently decalcified in 10 % ethylenediamine tetra acetic acid (EDTA), pH 7.2, and then embedded in paraffin. Six-micrometer sections were cut using a Leica RM 2255 microtome (Leica Microsystems, Richmond Hill, ON, Canada). After deparaffinization and hydration, the tissues were placed in a Tris-based antigen unmasking solution (Vector Laboratories, Burlington, ON, Canada) for 20 min at 92 °C, followed by blocking endogenous peroxidase with 1 % hydrogen peroxide for 10 min. Nonspecific binding was blocked by incubation in Tris-buffered saline containing 10 % normal goat serum for 20 min. Commercially available polyclonal rabbit antibodies tested were as follows: DKK1, FZD4, LRP5, LRP6, WNT4 and WNT10a (Abcam, Toronto, ON, Canada), PORCN (Novus Biologicals, Littleton, CO, USA), SOST (Abcam, Toronto, ON, Canada) as well as β-catenin (CTNNB1, Cell Signaling Technology, Danvers, MA, USA). A biotinylated goat anti-rabbit antibody (Vector Laboratories, Burlington, ON, Canada) was used as a secondary antibody (dilution 1:200) in 1 % normal goat serum. All sections were stained using the ABC avidin-biotin complex method (Vector Laboratories, Burlington, ON, Canada) followed by DAB peroxidase treatment (Vector Laboratories, Burlington, ON, Canada). Finally, all sections were counterstained with Mayer's hematoxylin (Sigma, Oakville, ON, Canada) and mounted with Permount (Fisher Scientific, Ottawa, ON, Canada). Multiple sections from each tissue type were used for immunohistochemistry. Photomicrographs of the tissues were taken using a Leica microscope (Leica Microsystems, Richmond Hill, ON, Canada) attached to a Q-Imaging camera (Olympus DP70, Concord, ON, Canada). For each experiment, we also included a negative control, where the same procedure was performed without the primary antibody.

### 2.5 Grading of the immuno-stained sections

Traditionally, immunohistochemistry is a semi-qualitative technique that is used for the anatomical identification of different cellular and extracellular components that stained positively. However, based on previously published reports (Tavakoli et al., 1999; Yeung et al., 2001), we have developed and used a semi-quantitative analysis to evaluate the images as previously described (Rauch et al., 2000; Hamdy et al., 2003; Haque et al., 2006). Briefly, the number of cells expressing the various proteins was assessed by cell counting. A blind observer graded sections as follows: 0, no staining; 1, staining in <25 % of cells; 2, staining in 25–50 % of cells; 3, staining in 50–75 % of cells; 4, staining in >75 % of cells. Chondrocytes (hypertrophic and proliferating), osteoblasts, osteoclasts, and osteocytes were identified morphologically. All analyses were performed separately for each slide. The grading was done blindly in two stages: the first analysis was made and then a second double check made by the same observer 3 weeks apart from the first analysis.

### 2.6 Statistical analysis

For the RNA seq data, the results are presented as group mean ± standard deviation (SD). Statistical significance was determined using the pairwise *t*-test for multiple comparisons. The significance for all tests was *p* < 0.05.

## 3 Results

### 3.1 Histology

**Fig. 1:**
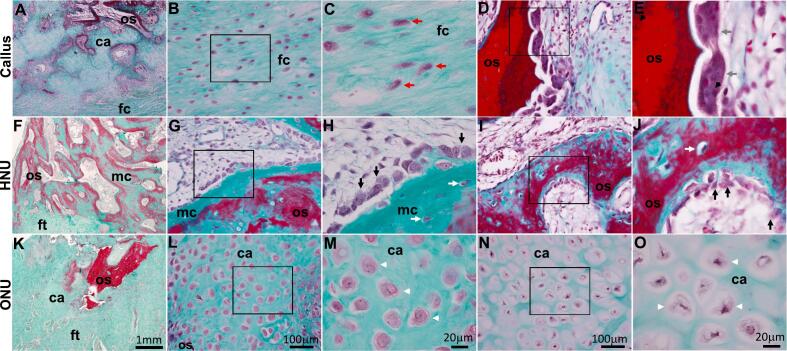
Fracture callus and nonunion tissue histology. Sections of fracture callus (A-E) and nonunion tissues, HNU (F-J) and ONU (K-O) were stained with Goldner's trichrome. C, H, M and E, J, O are enlarged images of areas shown in boxes in B, G, L, and D, I, N, respectively. Red arrows in C and grey arrows in E indicate fibroblasts, osteoclasts, respectively. Black arrows in H and J indicate osteoblasts. White arrows in H and J indicate osteocytes. White arrowheads in M and O indicate chondrocytes and hypertrophic chondrocytes, respectively. mc, mineralized callus; os, osteoid, ft., fibrous tissue, fc, fibrocartilage, ca, cartilage. Scale bar in A, F, K is 1 mm; B, D, G, I, L and N is 100 μm; and in C, H, M, E, J, and O is 20 μm.

**Fig. 2:**
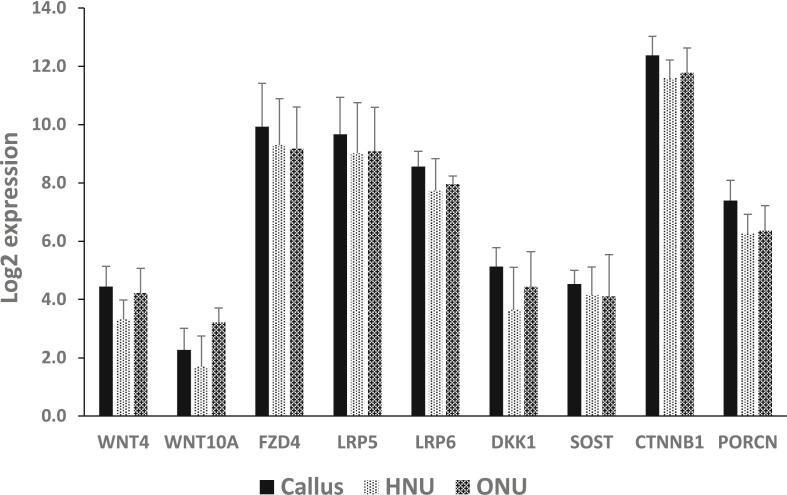
mRNA expression of Wnt signaling-related genes. The expression of Wnt ligands (WNT4, WNT10a), receptors (FZD4, LRP5, LRP6), inhibitors (DKK1, SOST) and modulators (CTNNB1 and PORCN) are presented as log2 expression as derived from RNA sequence data from our previous study (Salichos et al., 2024). No statistical significance was observed for any of these genes between any of the three tissue types.

The nonunion and callus sections were initially analyzed using standard light microscopy and Goldner's trichrome staining. In examining all samples, it was apparent that areas of new bone, fibrous tissue, cartilage, osteoid, mineralized callus was present indicating that all of the tissue processes that occur during fracture repair were represented. Also, due to different times of harvesting, several stages of tissue development were also present. In other words, not all of the calluses were exactly the same within each group of tissues (i.e. Callus or HNU or ONU). Regardless, Fig. 1 shows representative images of each tissue type. At low magnification, all of the aforementioned tissues types were present (Fig. 1A, F, K). At higher magnification, we observed fibrocartilage tissue (Fig. 1B and C), osteoblasts and osteocytes within mineralized osteoid (Fig. 1G, H, I, J), chondrocytes and hypertrophic chondrocytes within areas of cartilage (Fig. 2L, M, N, O), and osteoclasts (Fig. 1D and E). Again, we emphasize that these cells were found in all tissue types (Callus, HNU, ONU).

### 3.2 mRNA expression

RNA sequencing data revealed expression of all of the Wnt signaling-related genes tested in the three samples, Callus, HNU and ONU, but the expression level varied from gene to gene with CTNNB1 and displaying the highest levels (Fig. 2). The Wnt ligands, WNT4 and WNT10a displayed the lowest mRNA expression (Fig. 2). The inhibitors, DKK1 and SOST also displayed lower mRNA expression in comparison to the receptors and modulators (Fig. 2). Obviously, as these genes have different functions (e.g. secreted ligands vs. membrane receptors vs. inhibitors vs. modulators), it is not a surprise that their expression varied. Also, there was no statistically significant difference between the three samples for each gene, indicating that the expression was the same. Lastly, we also provide the expression of all of the Wnt signaling-related genes in Supplementary Fig. 1.

### 3.3 Expression of Wnt ligands (WNT4, WNT10a)

**Fig. 3:**
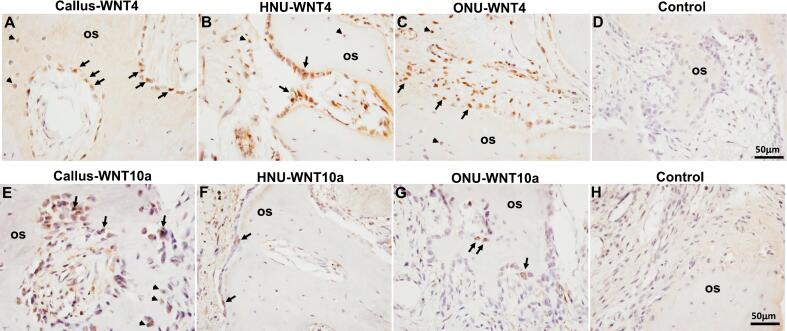
Wnt ligand expression. A-C indicates WNT4 expression in Callus, HNU and ONU, respectively. E-G indicates WNT10a staining in Callus, HNU and ONU, respectively. D and H represent negative controls (no primary antibody). Arrows in A, B, C, E, F, G all indicate positive staining in osteoblasts. Arrowheads in A, B, C, E all indicate positive staining in osteocytes. os, osteoid. Scale bar for all images is 50 μm.

For the spatial localization experiments, we selected representative Wnt-related proteins to examine; Wnt ligands (WNT4 and WNT10a), receptors (FZD4, LRP5, LRP6), inhibitors (DKK1, SOST) and modulators (CTNNB1 and PORCN). WNT4 expression was detected in all three tissues types, callus (Fig. 3A), HNU (Fig. 3B) and ONU (Fig. 3C). Specifically, WNT4 was strongly detected in osteoblasts present in the lining of the newly made woven bone in all three tissues (Fig. 3A-C). In addition, staining of WNT4 was also detected in osteocytes (Fig. 3A-C). Similarly, WNT10a exhibited the same pattern of expression, mostly in osteoblasts in all three tissues (Fig. 3E-G). In addition, we detected low expression of both WNT4 and WNT10a in osteoclasts (not in HNU), chondrocytes and hypertrophic chondrocytes (qualitative data now shown). The semi-quantitative analysis of all stained sections for these two proteins is shown in Table 2, indicating variable expression in the various cells of each tissue type, again indicating osteoblasts were the primary source of callus WNT4 and WNT10a. No expression of the proteins was detected in the negative control tissue sections (Fig. 3D and H).

**Table 2:** Immunohistochemistry results of WNT ligands, receptors, inhibitors, and modulators.

Category	Protein	Tissue	OT	OC	OB	C	HC
Wnt ligands	WNT4	Callus (N = 10)	1	1	2	1	1
		HNU (*N* = 7)	1	0	1	1	0
		ONU (N = 10)	1	1	2	1	1
	WNT10a	Callus (N = 10)	2	1	3	1	1
		HNU (N = 7)	1	0	2	1	0
		ONU (N = 10)	1	1	3	1	1
Wnt receptors	FZD4	Callus (N = 10)	1	1	2	1	1
		HNU (N = 7)	1	1	1	1	0
		ONU (N = 10)	1	1	1	1	0
	LRP5	Callus (N = 10)	1	1	1	1	1
		HNU (N = 7)	1	1	1	1	0
		ONU (N = 10)	1	2	1	1	1
	LRP6	Callus (N = 10)	2	2	2	1	1
		HNU (N = 7)	1	0	1	1	0
		ONU (N = 10)	1	1	1	1	0
Wnt inhibitors	DKK1	Callus (N = 10)	2	1	3	1	1
		HNU (N = 7)	1	0	2	1	0
		ONU (N = 10)	1	1	3	2	0
	SOST	Callus (N = 10)	3	1	1	0	1
		HNU (N = 7)	2	1	1	0	0
		ONU (N = 10)	3	1	1	1	0
Wnt modulators	CTNNB1	Callus (N = 10)	1	1	4	1	1
		HNU (N = 7)	1	0	2	0	0
		ONU (N = 10)	1	0	3	1	1
	PORCN	Callus (N = 10)	2	2	3	1	1
		HNU (N = 7)	1	1	2	1	0
		ONU (N = 10)	2	1	3	1	0

0, No positive staining; 1, <25 % of cells stained positive, 2, 25 to 50 % cells stained positive,

3, 50 to 75 % cells stained positive, 4, over 75 % cells stained positive.

**OT**: Osteocytes; **OC**: Osteoclasts; **OB**: Osteoblasts; **C**: Chondrocytes; **HC**: Hypertrophic chondrocytes.

### 3.4 Expression of Wnt receptors

**Fig. 4:**
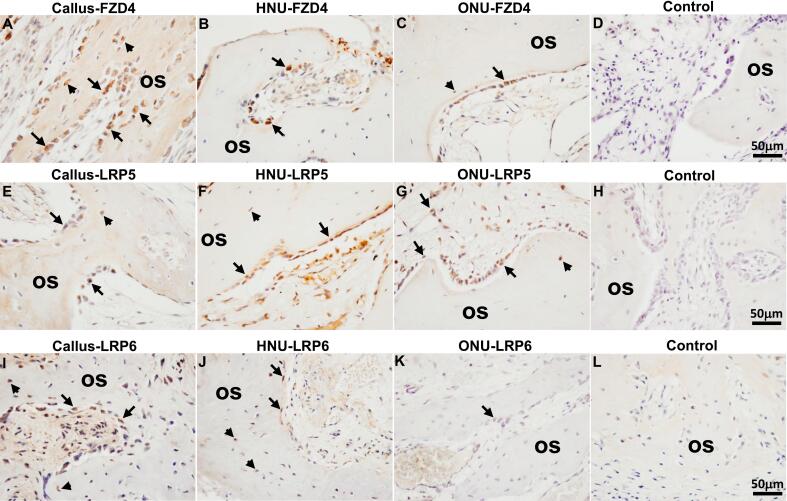
Wnt receptor expression. A, E, I indicate FZD4, LRP5 and LRP6 expression in Callus, respectively. B, F, J indicate FZD4, LRP5 and LRP6 expression in HNU, respectively. C, G, K indicate FZD4, LRP5 and LRP6 expression in ONU, respectively. D, H and L represent negative controls (no primary antibody). Arrows in A, B, C, E, F, G, I, J, K all indicate positive staining in osteoblasts. Arrowheads in A, C, E, F, G, I, J all indicate positive staining in osteocytes. os, osteoid. Scale bar for all images is 50 μm.

FZD4 expression was detected in all three tissue types, Callus (Fig. 4A), HNU (Fig. 4B) and ONU (Fig. 4C). Specifically, FZD4 was strongly detected in osteoblasts present in all three tissues around the lining of the newly made woven bone (Fig. 4A-C). In addition, staining of FZD4 was also detected in osteocytes, especially in the Callus tissue sample (Fig. 4A). LRP5 exhibited the same pattern of expression, mostly in osteoblasts as well as osteocytes in all 3 samples (Fig. 4E-G). LRP6 expression was detected at a lower level in osteoblasts in all three tissue types (Fig. 4I-K). In the semi-quantitative analysis of all stained sections for these three proteins (Table 2), we also detected low expression of FZD4 in osteoclasts (all three), chondrocytes (all three) and hypertrophic chondrocytes (only in callus). For LRP5, we detected low expression in osteoclasts (all three), chondrocytes (all three) and hypertrophic chondrocytes (not in HNU). With LRP6, we detected low expression in osteoclasts (not in HNU), chondrocytes (all three) and hypertrophic chondrocytes (only in callus). No expression of any of these proteins was detected in the negative control tissue sections (Fig. 4D, H, L).

### 3.5 Expression of Wnt-pathway inhibitors DKK1 and SOST

**Fig. 5:**
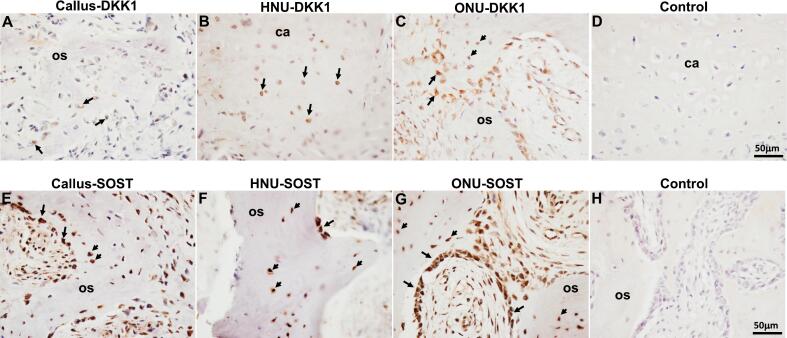
Wnt inhibitor expression. A-C indicates DKK1 expression in Callus, HNU and ONU, respectively. E-G indicates SOST staining in Callus, HNU and ONU, respectively. D and H represent negative controls (no primary antibody). Arrows in A, C, E, F, G all indicate positive staining in osteoblasts. Arrows in B indicate staining in chondrocytes. Arrowheads in C, E, F, G all indicate positive staining in osteocytes. os, osteoid, ca, cartilage. Scale bar for all images is 50 μm.

DKK1 expression was detected in all cell types in the callus (Table 2). Fig. 5A indicates DKK1 expression in callus osteoblasts. In HNU, we detected expression in osteocytes, osteoblast and chondrocytes (Table 2) and we show the chondrocyte expression (Fig. 5B). In ONU, we also detected expression in all cell types except hypertrophic chondrocytes (Table 2) and we show expression in osteoblasts and osteocytes (Fig. 5C). SOST exhibited robust expression in osteocytes and osteoblasts in all three tissue samples (Fig. 5E-G). Osteoclasts and cartilage cells showed the lowest expression (Table 2). Once again, no expression of any of these proteins was detected in the negative control tissue section (Fig. 5D, H).

### 3.6 Expression of Wnt modulators

**Fig. 6:**
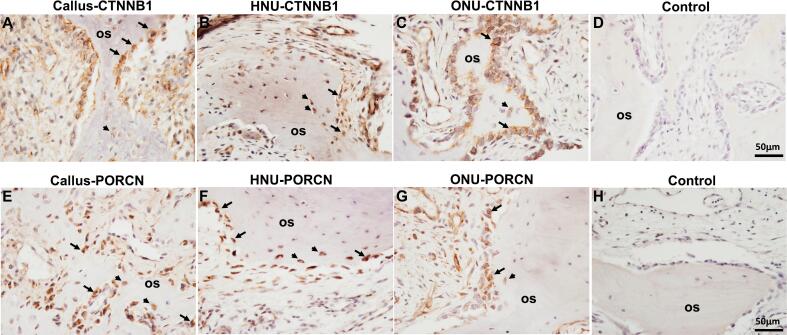
Wnt modulator expression. A-C indicates CTNNB1 expression in Callus, HNU and ONU, respectively. E-G indicates PORCN staining in Callus, HNU and ONU, respectively. D and H represent negative controls (no primary antibody). Arrows in A, B, C, E, F, G all indicate positive staining in osteoblasts. Arrowheads in A, B, C, E, F, G all indicate positive staining in osteocytes. os, osteoid. Scale bar for all images is 50 μm.

β-catenin expression was also very robust in all three tissue types, especially in osteoblasts (Fig. 6A-C). Osteocyte expression of β-catenin was also detected in all three tissues (Fig. 6A-C). Additionally, β-catenin expression, albeit, at lower levels, was observed in osteoclasts in the callus only, as well as in chondrocytes and hypertrophic chondrocytes (in Callus and ONU) (Table 2). PORCN expression was also very robust in osteoblasts and osteocytes in all three tissue types, present in the lining of the newly made woven bone (Fig. 6E-G). Expression in osteoclasts and chondrocytes was also detected in all tissues (Table 2) but only the callus displayed PORCN expression in hypertrophic chondrocytes. Lastly, no expression of any of the proteins was detected in the negative control tissue sections (Fig. 6D and H).

## 4 Discussion

The complexity of fracture healing has long been underestimated. Despite enormous efforts and robust animal research, so far, a single molecule, material or stimulus that determines the fracture healing process has remained elusive. This is not a surprise since fracture healing closely resembles embryonic skeletal development (Vortkamp et al., 1998; Ferguson et al., 1999) with thousands of genes activated (Hadjiargyrou et al., 2002; Joo et al., 2004). Adding to this complexity is also the differential expression of hundreds of regulatory RNAs which has also been reported (Hadjiargyrou et al., 2016; Hadjiargyrou and Komatsu, 2019; Xiong et al., 2020; Bourgery et al., 2021; Komatsu et al., 2021; Groven et al., 2022; Hadjiargyrou et al., 2023). Similarly, various signal transduction pathways are also activated during fracture healing, including, Wnt (Hadjiargyrou et al., 2002), BMP/TGF-b (Roberts and Ke, 2018), HIF-1α/VEGF (Komatsu and Hadjiargyrou, 2004; Komatsu et al., 2007), Notch (Wang et al., 2016), Hedgehog (Baht et al., 2014), etc. Control of signaling and subsequent gene and regulatory RNA expression is regulated by both, extracellular and intracellular signals (Hankenson et al., 2015; Papachristou et al., 2021; Rodríguez-Merchán, 2021). More importantly, we know that not all fractures heal normally and 2–5 % develop into a nonunion (Marsh, 1998). Therefore, understanding the molecular events of nonunions as compared to normal physiological fracture callus may enable us to identify molecular differences and/or similarities. As such, and given the significance of the Wnt signaling pathway in fracture healing (Schupbach et al., 2020), we wanted to directly compare Wnt signaling-related gene/protein expression between these tissues.

Our mRNA and immunohistochemical expression data presented herein are consistent with the findings of many other studies, predominantly in animals. That is, that Wnt activation occurs during human fracture repair as well. More importantly, Wnt activation is also robust in nonunion tissues at about the same level as physiological facture callus. Specifically, we detected strong expression of representative Wnt ligands, receptors, inhibitors and modulators, in both types of tissues. The highest expression was detected for β-catenin and the receptors while the ligands and inhibitors were expressed at a lower level. Our immunohistochemical data confirmed the mRNA results and showed strong β-catenin expression in osteoblasts consistent with the data from Zhong et al. (2006), Heilmann et al. (2013) and Haffner-Luntzer et al. (2021) who all reported its expression in post-fracture day 10 callus osteoblasts. β-catenin expression was also present in osteoblasts as early as post-fracture day 5 in mice (Kakar et al., 2007). We also observed low levels of β-catenin in chondrocytes, also consistent with previous reported data (Kakar et al., 2007; Kasaai et al., 2012; Haffner-Luntzer et al., 2021). Lastly, in a study where osteoblasts were isolated from endosteal cancellous bone fragments harvested from human hypertrophic nonunions, β-catenin was identified as one of the expressed genes (Hofmann et al., 2008).

As for the other Wnt modulator, PORCN, we were able to find only one study in the literature which reported that hematopoietic PORCN knockout mice had fewer fracture callus osteoclasts resulting in a delay of callus remodeling (Chua et al., 2021). Unfortunately, these authors did not present spatial expression of PORCN in the fracture callus. Regardless, our data show very strong expression of PORCN in osteoblasts and to a lesser degree, in osteoclasts and osteocytes in fracture callus. Lower expression of PORCN was also detected in the nonunion samples.

Previously, we showed that multiple members of the Wnt signaling pathway as well as some target genes were upregulated during 3–5 days after bone fracture and remained elevated, albeit at a lower level up to 21 days after the fracture (Hadjiargyrou et al., 2002; Zhong et al., 2006). Similarly, Chen et al. (2007) showed upregulation for WNT4, 5a, 5b, 11, and 13, FZD1, 2, 3, 5, 6, LRP6 and β-catenin mRNA within 1 week after fracture. Kakar et al. (2007) reported increased expression of WNT4, 5a, 5b, 10b, 11, LRP5 and LRP6 within 5 days after fracture in response to PTH, which remained elevated for 3 weeks. The same study also showed that DKK1 increased during the second week of fracture healing. We also showed an increased expression of WNT4 and WNT10a, FZD1 and 2, LRP5 and 6, β-catenin, DKK1, CTBP-1 and -2, sFRP-2 and -4 in a model of mouse distraction osteogenesis (Kasaai et al., 2012). Overall, there was increased expression of all these Wnt proteins during chondrogenesis, but they were downregulated at later stages of bone formation and calcification (Kasaai et al., 2012). Similarly, our data with the human samples show robust expression of Wnt receptors, FZD4, LRP5 and LRP6 in multiple callus cell types including osteoblasts, osteocytes, osteoclasts and chondrocytes. Their expression in hypertrophic chondrocytes was mainly found in the callus.

We also detected the expression of Wnt inhibitors, DKK1 and SOST predominantly in osteoblasts and osteocytes, respectively, in all three tissue types. Previously, Schwabe et al. (2014) used immunohistochemistry to detect various growth factors, including SOST in human atrophic nonunions. Another study also isolated a population of nonunion stromal cells also from atrophic nonunions and reported that the reduced capacity of these cells to form osteoblasts was associated with significantly elevated secretion of DKK1 (Bajada et al., 2009). Although, SOST is considered to be predominantly an osteocyte-specific protein, its expression was previously found in other cell types, including hypertrophic chondrocytes and cementocytes (Weivoda et al., 2017). Chen et al. (2013) also demonstrated that SOST is expressed in osteoblasts, consistent with our observation. Further, adding Wnt agonists or neutralizing antibodies to DKK1 or SOST were shown to improve bone healing by increasing the remodeling phase (Bao et al., 2017). Using the rat closed femoral fracture model in combination with inhibition of SOST and DKK1 via individual antibodies or a combination of the two, Florio et al., 2016, Florio et al., 2023 demonstrated significant increases in callus bridging, bone volume, and strength, thus improving fracture repair. Previously, Li et al. (2011) also showed increases in fracture callus mass and enhanced strength in SOST knockout mice. Another study reported that SOST knockout mice displayed increased trabecular bone volume at the fracture site and were biomechanical stronger (Alzahrani et al., 2016). Very recently, a human study reported that DKK1 levels in a fracture hematoma were significantly reduced while SOST levels significantly increased and that postoperatively, DKK1 peaked at week 2 while SOST levels were highest at week 8, convincing the authors to suggest that blocking both DKK1 and SOST could be therapeutically critical (Starlinger et al., 2024). But a previous clinical study of Romosozumab (an FDA-approved humanized monoclonal antibody SOST inhibitor) for the treatment of hip fractures did not improve fracture healing (Schemitsch et al., 2020). Lastly, we also reported RNA sequencing data showing the expression of all known Wnt ligands, receptors, inhibitors and modulators in HNU and ONU nonunions directly compared to physiological fracture callus (Salichos et al., 2024).

The early detection or better yet, prediction of a nonunion and subsequent surgical intervention will have a tremendous socioeconomic impact, not only for the patients, but for the society in general. Radiological markers have thus far failed to predict normal healing vs. disturbed healing. Hussein et al. (2018) recently used serum as a means of examining the progression of fracture repair over a course of 35 days in the hope of identifying potential markers that can coincide with the different biological stages of the repair process. They showed that peak levels for specific osteoblast related proteins appeared in the serum 3–4 days after their peak mRNA expression in callus. A list of 50 potential candidate biomarkers for fracture healing were identified, of which some are key members of the Wnt/β-catenin pathway and include, WIF-1, Wnt7A, DKK11 and DKK4. The authors also suggested that translational studies will be the only valid means to decide which of these proteins will be useful to predict disturbed healing (Hussein et al., 2018).

The crucial role of the canonical Wnt signaling in fracture healing has clearly been established in animal models. To our knowledge, this is the first report to document key members of the Wnt-pathway in normal callus and nonunion (HNU and ONU) tissues at both, the mRNA and protein levels (supporting our first hypothesis). Despite the strengths of our data (semiquantitative; human tissues, physiological callus, both classic nonunions types), there are some weaknesses which include the small number of patients, RNA from different specimens from patients used for histology and immunohistochemistry, various bone types, time since fracture, different sex or exactly matched patients. Unfortunately, these are some of the drawbacks related to human clinical studies.

Although, we did not find significant differences between the expression patterns in callus versus nonunion tissues (making our second hypothesis null) this is not a surprise given how critical the Wnt signaling pathway is to skeletal development (Teufel and Hartmann, 2019). Further, our data clearly confirms that Wnt signaling is a key process during human fracture healing and perhaps, having established this conclusively, physicians and scientists can start thinking about clinical application of Wnt signaling modulation and move beyond animal models. However, much remains to be elucidated as far as fine-tuning and cross talk with other known pathways such as BMP and TGF-β, before targeting Wnt-signaling in bone with small molecules becomes a promising approach to enhance fracture repair and prevent a nonunion. A number of different experimental approaches have emerged recently that seek to modulate the Wnt signaling pathway, including using Wnt ligands, miRNAs, targeting Wnt receptors, GSK-3β inhibitor, etc. (Schupbach et al., 2020). But with the widespread expression and various roles of the Wnt/β-catenin pathway, there is a high risk for off-target effects. Regardless, tremendous progress has been made in describing the functional role of Wnt signaling in fracture repair since it was originally described (Hadjiargyrou et al., 2002). We believe that future research aiming at determining the best approach(es) to activate the osteogenic potential of the Wnt pathway in the clinical management of nonunions is warranted.

The following is the supplementary data related to this article.

Supplementary Fig. 1 mRNA expression of Wnt signaling-related genes. The expression of Wnt family ligands (WNTs), receptors (LRPs and FZDs), as well as inhibitors (DKKs) are presented as log2 expression as derived from RNA sequence data from our previous study (Salichos et al., 2024). Supplementary Fig. 1

## CRediT authorship contribution statement

**Michael Hadjiargyrou:** Writing – review & editing, Writing – original draft, Supervision, Resources, Project administration, Investigation, Funding acquisition, Formal analysis, Conceptualization. **Maria Kotsiopriftis:** Methodology, Formal analysis, Data curation. **Dominique Lauzier:** Writing – original draft, Methodology, Formal analysis, Data curation. **Reggie C. Hamdy:** Writing – review & editing, Writing – original draft, Supervision, Resources, Formal analysis, Conceptualization. **Peter Kloen:** Writing – review & editing, Writing – original draft, Supervision, Resources, Project administration, Investigation, Conceptualization.

## Declaration of competing interest

None.

## Data Availability

Data will be made available on request.
