# Supplementary figures and images for: Activation of Wnt signaling in human fracture callus and nonunion tissues
Source: Bone Rep. 2024 Jun 19;22:101780. doi: 10.1016/j.bonr.2024.101780 (PMC11245924; doi:10.1016/j.bonr.2024.101780)

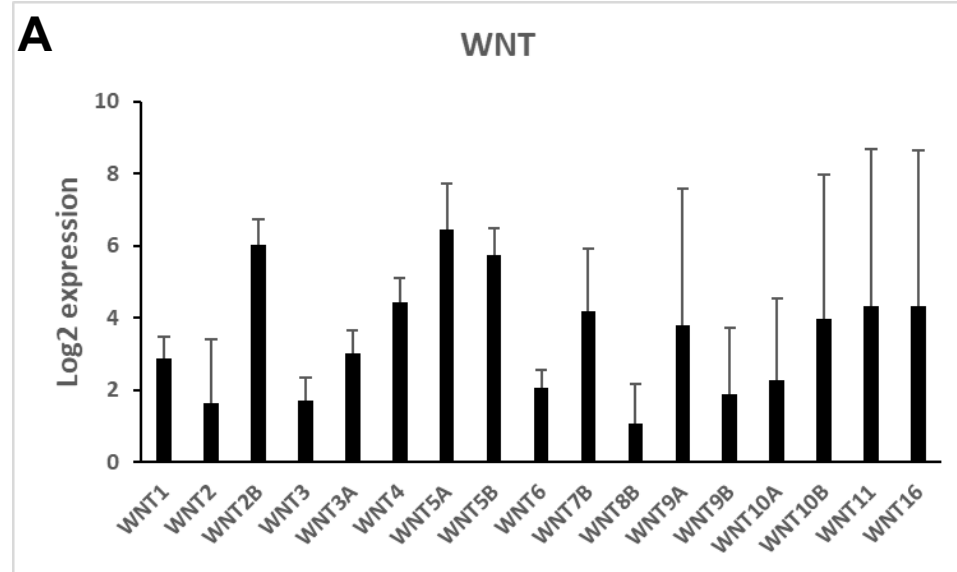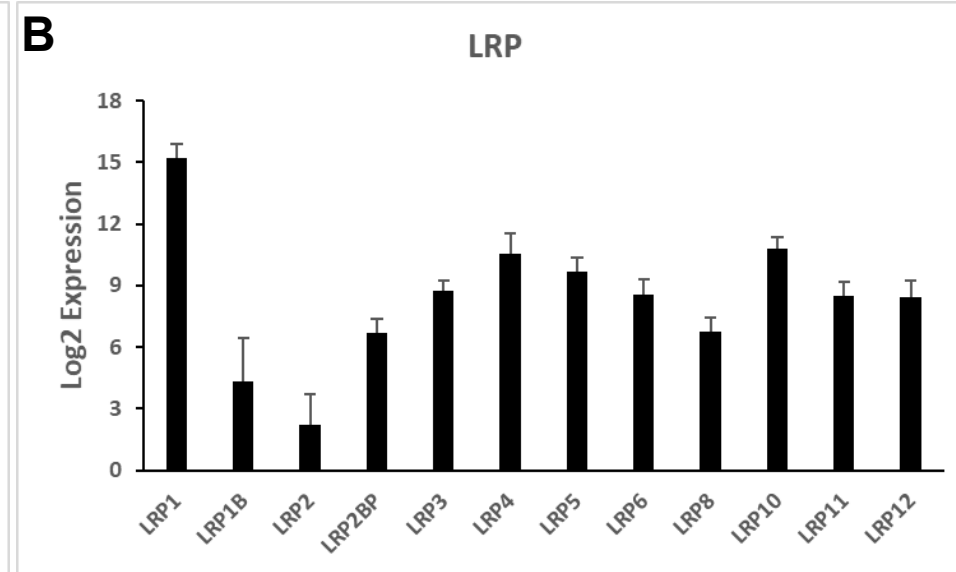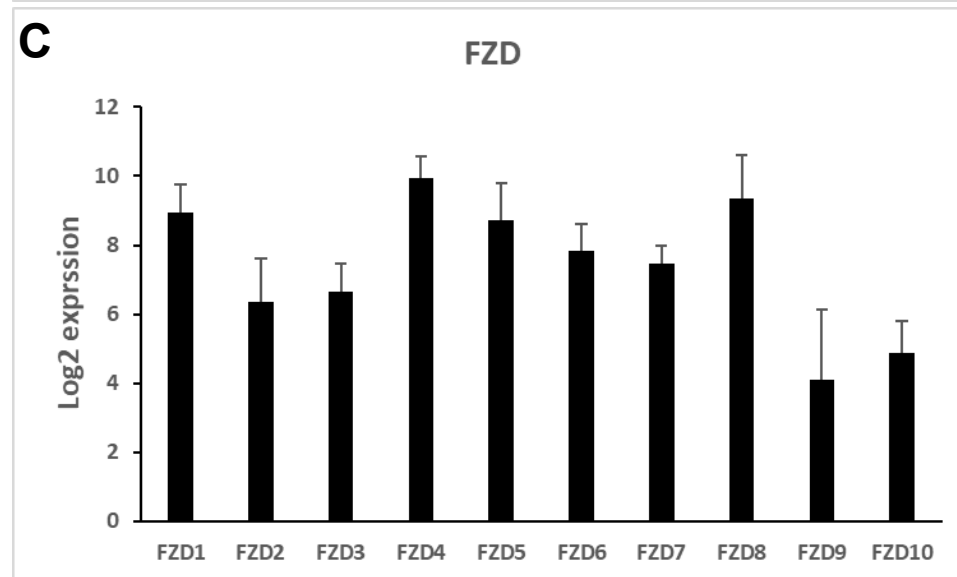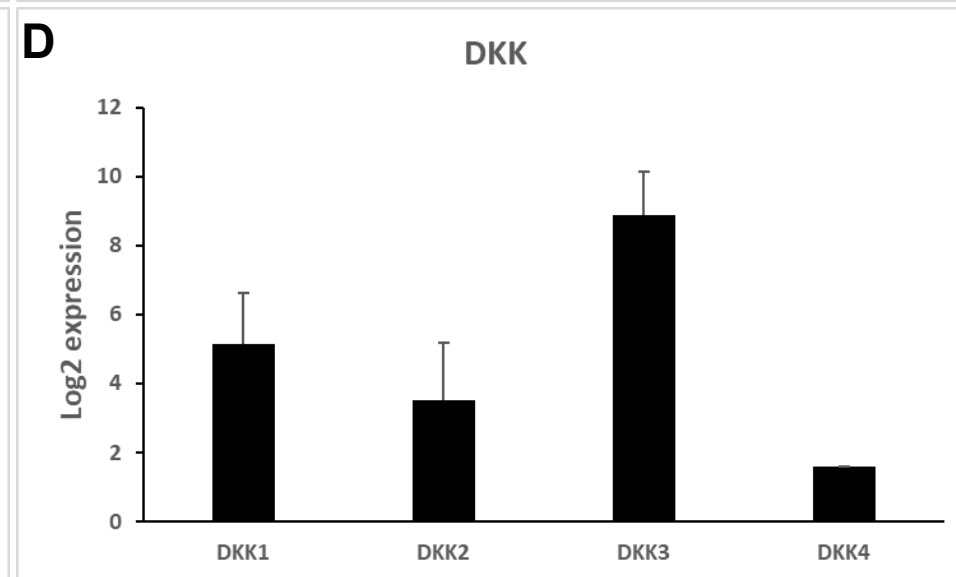

Supplement: Supplementary Fig. 1 — mRNA expression of Wnt signaling-related genes. The expression of Wnt family ligands (WNTs), receptors (LRPs and FZDs), as well as inhibitors (DKKs) are presented as log2 expression as derived from RNA sequence data from our previous study (Salichos et al., 2024). [file mmc1.pdf]
